# News from Societies

**Published:** 1953

**Authors:** 


### *News from Societies*

### BRISTOL MEDICO-CHIRURGICAL SOCIETY

The Annual General Meeting was held on October 14th in the Physics [illegible] Theatre at the Royal Fort, and was attended by over 135 members and guests.

After the Minutes of the previous meeting had been read and approved, the Pres[illegible] Dr. G. E. F. Sutton, M.C., spoke of the deaths during the last year of Pro[illegible] Rendle Short, Mr. A. J. Wright, and Dr. W. V. Woods. Members stood for a [illegible] time as a token of respect.

The President invested his successor, Mr. R. Ramsay Garden, M.A., M.B., D.O.M.S., who gave his Presidential Address on the subject of " Blindness ", which is to be reported in full in the *Medical Journal of the South West*. A vote of thanks for the Presidential Address was proposed by Mr. Jackman and seconded by Mr. Palin.

The meeting then proceeded to elect the following officers:

*President-Elect:* Dr. Gordon Heron. *Hon. Secretary:* Dr. Brian McConnell. *Hon. Treasurer:* Dr. Macrae. *Hon. Medical Librarian:* Dr. Gale. *Three Members of Committee:* Dr. J. M. Naish and Messrs. Angell James and H. D. Fairman. *Two Members to serve on University Library Sub-Committee:* Mr. Lucas and Dr. Marwood.

In his Annual Report the Hon. Secretary was able to report a most successful year with a very large increase in membership.

The Hon. Treasurer gave a reassuring account of the finances of the Society.

The Hon. Editor of the *Journal* reported certain difficulties which had been met in the past year—causing delay in the production, but gave promise of a remedy of this before many months.

Meetings are usually held at the Royal Fort on the second Wednesday of each month at 8.15 p.m. The following tentative programme has been arranged for the coming session and exact details of subject will be announced in notices sent out before meetings:

November 11th: Clinical meeting at the Bristol Royal Infirmary.

December 9th: R. V. Cooke, Esq., CH.M., F.R.C.S., on malignant disease of the bowel.

January 13th: G. M. Fitzgibbon, Esq., M.D., F.R.C.S., on a subject connected with plastic surgery.

February 10th: Sir G. R. McRobert, C.I.E., M.D., F.R.C.P., on a subject connected with tropical medicine.

March 10th: Dr. Beryl Corner and Dr. A. M. G. Campbell on meningitis.

April 14th: Dr. G. F. Abercrombie on a subject connected with general practice.

May 12th: Ashton Miller, Esq., M.D., F.R.C.S., and J. P. Mitchell, Esq., M.B., F.R.C.S., on a subject connected with urological surgery.

### CLINICAL SOCIETY OF BATH

*President:* Dr. G. D. Kersley.

*Hon. Secretary:* Dr. D. W. Pugh, D.S.C., " Dunedin Lodge ", Weston Road, Bath (Tel. No. 78366).

Meetings are held at the Royal United Hospital, Bath, on the second Friday of each month with cases at 8 p.m. and discussion at 8.30 p.m.

November: Dr. J. R. O'Neill on **"Generalized Lymphadenopathy"**.

### PLYMOUTH MEDICAL SOCIETY

*President:* Mr. C. S. C. Prance.

*Hon. Secretaries:* Mr. W. B. Waterfall, 3 Hartley Avenue, Plymouth; Dr. J. W. M. Owen, Castlehayes, Plympton.

Future meetings:

November 20th: Dr. P. M. F. Bishop on " **Endocrine Therapy** ".

January 15th, 1954: Mr. A. L. Rowse on " **Elizabethan England and To-day** ".

March 5th, 1954: Sir Russell Brain, P.R.C.P., on " **Cervical Spondylosis** ".

### DEVON AND EXETER MEDICO-CHIRURGICAL SOCIETY

A meeting of the Society was held at the Princess Elizabeth Orthopaedic Hospital, Exeter, on April 19th, 1953.

After a brief introduction by Mr. Norman Capener, a number of patients were presented by the surgical staff of the Hospital.

**Mr. Durbin** showed a patient who had recovered from an almost complete pseudo-paralysis due to calcinosis; the extensive infiltration of all muscles with deposits of calcium had resolved by the extrusion of the material through multiple skin sinuses. This patient had been shown to the Society four years previously when completely bedridden and unable to feed herself. He also discussed the possibilities of r[illegible] structive surgery in poliomyelitis. Apart from the first, these cases and all others s[illegible] were ordinary in-patients of the hospital at the time of the meeting, and they de[illegible] strated the range of conditions under treatment.

**Mr. Blundell Jones** discussed the treatment of paralytic dislocations of the h[illegible] children, secondary to poliomyelitis or to cerebral palsy. The combination of dislo[illegible] hips and deficient musculature made walking impossible; the attainment of s[illegible] reductions might allow the deficient muscles to develop sufficient control to enabl[illegible] child to walk with the aid of appliances. If the condition was recognized early en[illegible] the simple procedure of trochanteric ostectomy would stabilize the hips.

The Registrars, **Mr. Merryweather** and **Mr. Robins,** showed patients with i[illegible] vertebral disc protusion, rheumatoid arthritis and fracture of the talus. Only a small proportion of patients in the first group came to operation, the majority respo[illegible] to conservative treatment. The value of a plaster jacket was emphasized; for all s[illegible] conditions the total applied in the hospital out-patient department averaged [illegible] a month.

Rheumatoid arthritis needed co-operation between physician and surgeon. I[illegible] early stages, pain could be relieved and deformity prevented by splinting joints i[illegible] physiological position and controlling their movement. In the late stages ope[illegible] was sometimes indicated to give stability or to correct deformity.

With severe fracture-dislocations of the talus-the urgent necessity was to re[illegible] tension of the overlying soft tissues which jeopardized the viability of the overlying [illegible] and of the foot as a whole.

The Resident Staff then took part in the demonstration. **Mr. Page** showed chil[illegible] with structural scoliosis. The severe scoliosis, especially if developing after an a[illegible] of poliomyelitis in infancy, presented one of the most difficult problems in orthopae[illegible] Three children were shown, each of whom had recently undergone correction foll[illegible] by an operation for spinal fusion, when aged about twelve.

In the treatment of osteomyelitis, **Mr. Carrier** stressed the importance of early o[illegible] tion if there were not a rapid response to conservative measures. Antibiotics di[illegible] reach a focus of infection when the local blood supply was cut off; with the preval[illegible] of resistant strains it was necessary to obtain the organism in order to determin[illegible] sensitivity to the various antibiotics.

**Miss Matthews** illustrated the conservative treatment of congenital dislocatio[illegible] the hip by presenting children on abduction frames, in plaster and walking in [illegible] Browne splints.

After these patients had been shown to the meeting, the Secretary, after than[illegible] Mr. Capener and the members of the staff of the hospital, asked whether there [illegible] one principle which the orthopaedic unit at Exeter tried in particular to teach. [illegible] Capener thought that eclecticism was most important. For example, at least [illegible] types of operation were commonly performed for osteoarthritis of the hip, the proce[illegible] being carefully chosen which would best suit the individual need.

### SOUTH SOMERSET MEDICAL CLUB

A meeting of the Club was held at Yeovil on September 17th, 1953. The Club [illegible] addressed by Dr. C. de W. Kitcat on " The treatment of the tuberculous patie[illegible] home ".

The next meeting of the Club will be held at Yeovil on November 19th, 1953, [illegible] the address will be given by Dr. Gardiner-Hill whose subject will be the " Ad[illegible] Gland ".

### THE SOUTH WEST REGIONAL PAEDIATRIC CLUB

A meeting was held at Cheltenham on May 16th and 17th, 1953. Cases were [illegible] sented by Dr. C. P. Donnison and Dr. A. F. D. Darlington. A demonstratio[illegible] Orthoptic cases was given by Mr. J. G. D. Currie and Mr. R. W. Stephenson, [illegible] discussion on the scope and functions of a Children's Orthoptic Department.

Papers were read by Dr. C. P. Donnison, " Congenital Atresia of the Oesophag[illegible]

Dr. J. A. Crawford, " Some Psychosomatic Syndromes in Child Guidance Practice "; and Dr. Beryl Corner, " Retrolental Fibroplasis ".

1953 TORQUAY AND DISTRICT MEDICAL SOCIETY

October 8th: Dr. Alice Stewart, F.R.C.P., " **The Day-to-Day Work of a University Department of Social Medicine** ".

Dinner and Dance at the Palace Hotel, Torquay.

November 12th: Sir Zachary Cope, " **The Birth of Modern Scientific Medicine** ".

November 26th: Clinical Discussion, " **Is the Health Visitor really necessary?** "

December 10th: Dr. G. H. Tovey, " **Salt and Water in Clinical Medicine** ".

1954

January 14th: Clinical Meeting in conjunction with S.W. Branch of the British Medical Association.

January 28th: Dr. Margaret Jackson, " **Investigation and Treatment of Subfertile Marriages with special reference to the Post-Coital Test** ".

February 25th: Professor Bruce Perry, " **Rheumatic Heart Disease** ".

March 26th: Dr. F. E. Camps, " **Death at Rillington Place** ".

April 22nd: Dr. Hugh Jolly, " **Common Paediatric Disorders in General Practice** ".

June 3rd: Summer Meeting at St. Loyes College, Exeter.

